# Probiotic research in neonates with congenital gastrointestinal surgical conditions – Now is the time

**DOI:** 10.1111/1751-7915.13358

**Published:** 2018-12-21

**Authors:** Shripada C. Rao, Sanjay K. Patole

**Affiliations:** ^1^ Neonatal Intensive Care Unit Perth Children's Hospital Hospital Avenue Nedlands WA 6009 Australia; ^2^ Centre for Neonatal Research and Education University of Western Australia Perth WA Australia; ^3^ Neonatal Directorate King Edward Memorial Hospital for Women Perth WA Australia

## Abstract

Neonates with congenital gastrointestinal surgical conditions (CGISC) receive parenteral nutrition, get exposed to multiple courses of antibiotics, undergo invasive procedures, and are nursed in intensive care units. They do not receive early enteral feeding and have limited opportunities for skin to skin contact with their mothers. Many of these infants receive gastric acid suppression therapies. All these factors increase the risk of gut dysbiosis in these infants. Gut dysbiosis is known to be associated with increased risk of infections and other morbidities in ICU patients. Experimental studies have shown that probiotics inhibit gut colonization with pathogenic bacteria, enhance gut barrier function, facilitate colonization with healthy commensals, protect from enteropathogenic infection through production of acetate, reduce antimicrobial resistance, enhance innate immunity, and increase the maturation of the enteric nervous system and promote gut peristalsis. Through these mechanisms, probiotics have the potential to decrease the risk of sepsis and inflammation, improve feed tolerance and minimise cholestasis in neonates with CGISC. Among preterm non‐surgical infants, evidence from more than 35 RCTs and multiple observational studies have shown probiotics to be safe and beneficial. A RCT in neonates (N=24) with gastroschisis found that probiotic supplementation partially attenuated gut dysbiosis. Two ongoing RCTs (total N=168) in neonates with gastrointestinal surgical conditions are expected to provide feasibility data to enable the conduct of large RCTs. Rigorous quality assurance of the probiotic product, ongoing microbial surveillance and clinical vigilance are warranted while conducting such RCTs.

The major congenital gastrointestinal surgical conditions (CGISC) include oesophageal atresia, gastroschisis, exomphalos, malrotation and volvulus, duodenal atresia, intestinal atresia, meconium ileus, hypoplastic colon, meconium peritonitis, intestinal stenosis, congenital short bowel syndrome, Hirschsprung disease (HD), anorectal malformations and others. In addition to surgical repair, strategies for managing such conditions include early commencement of enteral feeds, standardization of feeding advancement, strict hand hygiene and aseptic precautions for indwelling catheters (Graham, 2010; Lauriti *et al*., 2014; Savoie *et al*., 2016; Dama *et al*., 2017). Despite such best practices and advances in surgical techniques, morbidities including feed intolerance, healthcare‐associated infections, cholestatic jaundice, growth failure and neurodevelopmental disabilities continue to impose significant health burden on this cohort (Willis *et al*., 2010; Bishay *et al*., 2012; Wang *et al*., 2014; Dwyer *et al*., 2016; Hong *et al*., 2018). Additional strategies are hence required to improve their outcomes.

## Gut dysbiosis in infants with CGISC

Neonatal gut microbiota develops rapidly after birth and achieves an adult‐like composition and stability by 2–3 years of age (Arrieta *et al*., 2014). The evolution of gut microbiome is affected in infants with CGISC admitted in intensive care units (ICUs). These infants receive parenteral nutrition (PN), get exposed to multiple courses of antibiotics, do not receive early enteral feeding and optimal maternal skin to skin contact. Decontamination of the skin for surgery, exposure to gastric acid suppressants, breakdown of natural barriers due to invasive procedures and indwelling tubes and catheters, colonization of the ICU room surfaces and hands of the healthcare providers also contribute to the risk of gut dysbiosis in infants with CGISC (Donnell *et al*., 2002; van Saene *et al*., 2003; Hussey *et al*., 2011; Fouhy *et al*., 2012; Ralls *et al*., 2016; Rogers *et al*., 2016; Kitsios *et al*., 2017).

- *PN and gut dysbiosis:* The role of PN in gut dysbiosis deserves attention as it is often the main/only source of nutrition in infants with CGISC. Lavallee *et al*. (2017) randomized neonatal piglets to receive total parenteral nutrition (TPN) or sow feeds (SF) for 14 days. Ileal segments and mucosal scrapings were used to assess the microbiota composition by 16S rRNA gene sequencing. Significant dysbiosis was noted in the TPN group, especially in those which received soy‐based lipids. In another study, using a mouse model, Ralls *et al*. (2016) reported permeation of TPN‐derived nutrients into the gut lumen, where they were preferentially utilized by Enterobacteriaceae, which then flourished.
- *Antibiotics and gut dysbiosis:* Fouhy *et al*. (2012) compared the gut microbiota of nine newborn infants treated with parenteral ampicillin and gentamicin, with that of nine matched healthy infants. Gut microbiota of the antibiotic‐treated infants showed significantly higher proportions of Proteobacteria and lower proportions of Actinobacteria and the associated genus Bifidobacterium, as well as the genus Lactobacillus compared with the untreated controls 4 weeks after the cessation of treatment. Even by week 8, Proteobacteria levels remained significantly higher in the treated infants (Fouhy *et al*., 2012). Increased abundance of Proteobacteria is a concern because it is considered as a potential diagnostic signature of dysbiosis and risk of disease (Shin *et al*., 2015).
- *The ICU ecosystem and gut dysbiosis:* In a study in adult ICU patients, McDonald *et al*. (2016) showed evidence of extreme dysbiosis. The phylogenetic diversity at discharge was significantly lower than at admission. Faecal samples tended to have a lower relative abundance of Firmicutes and Bacteroidetes and an increased relative abundance of Proteobacteria and well‐recognized pathogens such as Enterobacter and Staphylococcus (McDonald *et al*., 2016). In a study in paediatric ICUs, Rogers *et al*. (2016) reported taxonomic alterations in the gut microbiota. These included enrichments of gut pathogens such as Enterococcus and Staphylococcus at multiple body sites and depletion of commensals such as Faecalibacterium and Ruminococcus from stool samples. Alpha and beta diversity were unstable over time (Rogers *et al*., 2016).

Studies have shown an association between gut dysbiosis and morbidities such as hospital‐acquired infections in neonates with surgical conditions (Donnell *et al*., 2002; van Saene *et al*., 2003) and Hirschsprung‐associated enterocolitis (HAEC) (Li *et al*., 2016).

## Probiotics for CGISC

Given that gut dysbiosis occurs and is associated with morbidities in infants with CGISC, optimization of gut microbiota by probiotics is a potentially beneficial strategy to improve their outcomes.

Probiotics are defined as live microorganisms that when administered in adequate amounts confer health benefits on people with specific illnesses (Hill *et al*., 2014). Probiotics inhibit gut colonization with pathogenic bacteria (Sassone‐Corsi and Raffatellu, 2015), enhance gut barrier function (Bron *et al*., 2017), facilitate colonization with healthy commensals (Garrido *et al*., 2012), protect from enteropathogenic infection through production of acetate (Fukuda *et al*., 2011), reduce antimicrobial resistance (Taft *et al*., 2018), enhance innate immunity (Giorgetti *et al*., 2015) and increase maturation of the enteric nervous system and promote gut peristalsis (Hyland and Cryan, 2016; De Vadder *et al*., 2018). Through these mechanisms, probiotics have the potential to decrease the risk of sepsis, improve feed tolerance and minimize parenteral nutrition‐associated cholestasis in infants with CGISC.

- *Evidence from studies in adult patients:* A recent meta‐analysis of 20 RCTs (*N* = 1374) concluded that probiotic/symbiotic supplementation decreases the risk of surgical site and urinary tract infections in patients undergoing abdominal surgery (Lytvyn *et al*., 2016). Another meta‐analysis that included 28 RCTs (*n* = 2511) involving adult patients undergoing gastrointestinal surgery came to similar conclusions (Yang *et al*., 2017). The durations of hospital stay and antibiotic therapy were shorter in the probiotics/symbiotic group vs controls (Yang *et al*., 2017). The need for caution in interpreting the results was emphasized considering the high risk of bias in included studies (Lytvyn *et al*., 2016; Yang *et al*., 2017).
- *Evidence from studies in paediatric patients:* In a RCT, 30 children (<15 years) with various surgical (majority gastrointestinal) conditions were supplemented with probiotic *Bifidobacterium breve* BBG‐01 or placebo daily from 7 days before the surgery until discharge. Probiotic supplementation was safe. It improved the gut flora, increased the concentration of faecal acetic acid and decreased the risk of septicaemia (Okazaki *et al*., 2016). A recent meta‐analysis that included 198 infants with HD (two RCTs, three observational studies) reported that the incidence of HAEC 22.6% in the probiotic group vs. 30.5% in the controls, but the difference was not statistically significant (OR 0.72; 95% CI 0.37–1.39; *P*  = 0.33; Nakamura *et al*., 2018). Majority of the infants in the included studies were outside the neonatal period.
- *Evidence from studies in neonates*: A systematic review (Rao *et al*., 2018) that focussed on CGISC exclusively in the neonatal population found only two small RCTs (Murakami *et al*., 2016; Powell *et al*., 2016). The Powell *et al*. (2016) RCT included 24 neonates with gastroschisis (Probiotics: 12, Placebo: 12). The probiotic supplement was administered for 6 weeks or until hospital discharge, whichever came first. Significant dysbiosis was noted in the study infants, and it was partially attenuated by administration of *Bifidobacterium longum* subsp. infantis (Powell *et al*., 2016). In the RCT by Murakami *et al*. (2016), four surgical neonates (duodenal atresia, anorectal malformations) received probiotics, four received no probiotics. Bifidobacteriaceae was more abundant in neonates who had not received probiotics. It was concluded that surgical stress appeared to affect the intestinal microbiota considerably. The need for further RCTs in this area was emphasized.

## Safety of probiotics

Evidence from over 35 RCTs with a total sample size of nearly 12 000 and observational studies with over 14 000 participants show that probiotics are beneficial and safe in preterm non‐surgical infants (Olsen *et al*., 2016; Rao *et al*., 2016; Sawh *et al*., 2016; Dermyshi *et al*., 2017). Even a large RCT that did not show benefits of probiotic supplementation acknowledged that short‐term safety of probiotics was good in preterm infants (Costeloe *et al*., 2016). Recent meta‐analyses have shown that probiotics do not increase or decrease the risk of intraventricular haemorrhage, chronic lung disease, retinopathy of prematurity and neurodevelopmental outcomes in preterm non‐surgical infants (Cavallaro *et al*., 2017; Villamor‐Martinez *et al*., 2017; Upadhyay *et al*., 2018).These findings provide reassurance regarding medium‐term safety of probiotics in preterm infants. However, there are few case reports of sepsis due to probiotic organisms (Ohishi *et al*., 2010; Vallabhaneni *et al*., 2015; Brecht *et al*., 2016). Hence, constant vigilance and quality assurance of the product while conducting RCTs of probiotic supplementation in infants with CGISC are warranted.

## Ongoing RCTs of probiotics in infants with CGISC

To our knowledge, currently, there are two ongoing RCTs evaluating the role of probiotics in this area. One trial is being conducted in Calgary (Canada) and aims to recruit 88 infants born between 23 and 42 weeks of gestation who require gastrointestinal surgery (Mugarab‐Samedi *et al*., 2017). The probiotic supplement is FloraBabyTM (Renew Life Canada, Oakville, ON, Canada). Each sachet (1 g) will have 4 billion colony‐forming units (CFU) of probiotics, consisting of *Bifidobacterium breve* (HA‐129), *Lactobacillus rhamnosus* (HA111), *Bifidobacterium bifidum* (HA‐132), *Bifidobacterium longum* subsp. infantis (HA‐116) and *Bifidobacterium longum* subsp. longum (HA‐135). Placebo is maltodextrin. The primary outcome of interest is length of hospital stay. Stool microbial analysis using culture independent 16S rRNA studies will be undertaken.

The other study (ours) is being conducted in Western Australia (Rao *et al*., 2017). Sixty infants (≥35 weeks’ gestation) with major CGISC will be recruited. The probiotic group will receive 3 × 10^9^ CFU/day (i.e. 3 billion organisms) in 1.5 ml of the expressed breast milk or sterile water, given as a single daily dose via the orogastric/nasogastric feeding tube or orally. The probiotic sachet (Morinaga Industries, Tokyo, Japan) will contain a mixture of three strains (*B. breve* M‐16V, *B. longum* subsp. infantis M‐63 and *B. longum* subsp. longum BB536 (1 × 10^9^ CFU of each strain per 1 g sachet). Placebo is maltodextrin. Supplementation will be commenced as soon as possible after admission once the baseline stool samples are collected and will be continued until discharge. Primary outcome will be gut microbiota (using 16 s ribosomal RNA Pyrosequencing studies for phylogenic profiling) on stool samples. Secondary outcomes will be stool short‐chain fatty acids and relevant clinical outcomes.

## Conclusions

In summary, probiotic supplementation has the potential to minimize gut dysbiosis and improve clinical outcomes of neonates with CGISC. Though small, the completed and ongoing RCTs will provide important data and confidence to embark on adequately powered large RCTs in this exciting area.

## Conflict of interest

None declared.
